# Epidemiology of Porcine Circovirus Type 2 Circulating in Wild Boars of Portugal during the 2018–2020 Hunting Seasons Suggests the Emergence of Genotype 2d

**DOI:** 10.3390/ani12040451

**Published:** 2022-02-12

**Authors:** Alícia de Sousa Moreira, Sérgio Santos-Silva, João Mega, Josman D. Palmeira, Rita T. Torres, João R. Mesquita

**Affiliations:** 1 ICBAS—School of Medicine and Biomedical Sciences, Porto University, 4050-313 Porto, Portugal; up201904302@edu.icbas.up.pt (A.d.S.M.); up202110051@edu.icbas.up.pt (S.S.-S.); joaomega@outlook.com (J.M.); 2 Department of Biology & CESAM, University of Aveiro, Campus de Santiago, 3810-193 Aveiro, Portugal; josmandantasp@gmail.com (J.D.P.); rita.torres@ua.pt (R.T.T.); 3 Epidemiology Research Unit (EPIUnit), Instituto de Saúde Pública da Universidade do Porto, 4050-600 Porto, Portugal; 4 Laboratório Para a Investigação Integrativa e Translacional em Saúde Populacional (ITR), 4050-313 Porto, Portugal

**Keywords:** PCV-2, wild boar, wildlife, emerging infectious diseases

## Abstract

**Simple Summary:**

Porcine circovirus type 2 (PCV-2) is a swine disease associated with post-weaning multi-systemic wasting syndrome, which causes a high economic impact on animal production. Recently, new evidence has suggested the increasing circulation of the PCV-2d genotype. We screened wild boar stools collected from several districts across Portugal during the 2018–2020 hunting seasons, for PCV-2 and genetically characterized detected strains. From a total 76 stool samples of wild boar tested, two sequences were obtained, both of the PCV-2d genotype, showing the presence of this genotype in Portugal for the first time. Monitoring wild PCV-2 reservoirs is important for both veterinary public health and economic reasons.

**Abstract:**

Porcine circovirus type 2 (PCV-2) is associated with several syndromes affecting swine, also known as porcine-circovirus-associated diseases, of which post-weaning multi-systemic wasting syndrome stands out due to its high economic impact on swine production. Recent data suggest the increasing circulation of the PCV-2d genotype in several countries worldwide. To provide updated data on PCV-2 genotypes currently circulating in swine in Portugal, we screened wild boar stools collected from several districts across Portugal, during the 2018–2020 hunting seasons, for PCV-2 and genetically characterized detected strains. From a total of 76 stool samples of wild boar tested by PCR for the partial PCV-2 *ORF2* gene, two sequences were obtained (2/76; 2.6%, 95% confidence interval: 0.032–9.18). Bidirectional sequencing showed that the sequences were 100% identical and both of the PCV-2d genotype, showing for the first time the presence of this genotype in Portugal. Monitoring wild PCV-2 reservoirs is important for both veterinary public health and economic reasons, since PCV-2 infection has a strong economic impact on the swine industry.

## 1. Introduction

Porcine circoviruses (PCV) are members of the Circoviridae family and are small (16–18 nm diameter), non-enveloped viruses with a spherical shape and single-stranded DNA genome [1]. As of now, four genetically distinct PCVs (PCV-1–4) have been reported. PCV-1 is considered apathogenic, and the roles of PCV-3 [2] and PCV-4 [3] and their potential impact in disease are still not clarified. However, unlike the previous types, PCV-2 is clearly associated with several syndromes affecting domestic and wild swine, also known as porcine-circovirus-associated diseases. Among these, post-weaning multi-systemic wasting syndrome (PMWS) is of particular relevance due to its economic impact on swine production [4,5], with estimated costs in the European Union between EUR 562 and EUR 900 million per year [6]. In 2008, the economic impact of PCV-2 in England was estimated at GBP 52.6 million per year, demonstrating the substantial economic impact of this virus on domestic swine [7]. As of now, four different PCV-2 genotypes (PCV-2a, PCV-2b, PCV-2c, and PCV-2d) have been identified. Recent data point towards the increasing circulation of the latter genotype in many countries, most likely replacing previously circulating strains [8].

PCV-2 has a circular genome with 1766–1768 nucleotides organized in two open reading frames, namely the *ORF1* (rep gene), encoding two proteins associated with replication; and *ORF2* (cap gene), encoding the capsid protein [9]. Interestingly, of the single-stranded DNA viruses, PCV-2 is known for its substantial genetic diversity, with a high nucleotide substitution rate) almost equal to that of RNA viruses [1]. Due to its high transmission rate, PCV-2 infection is widespread in countries with a strong swine industry, posing significant veterinary public health issues [4]. Likewise, it was confirmed that wild boars are susceptible to PCV-2 infection and viral circulation in European populations. Some data report circulation in countries such as Germany (18.1%) [10], Belgium (35.6%) [11], Slovenia (25%) [12], the Czech Republic (43%) [13], Italy (10.9%) [14], Spain (23–58%) [15] and Hungary (20.5%) [16].

To the best of the authors’ knowledge, only one study dating from 2011 reports the occurrence of PCV-2 in domestic pigs in Portugal [17]. In that study, the PCV-2b genotype was shown to be dominant and likely introduced to Portugal around 2003, circulating since then. The authors also report the potential introduction of genotype PCV-2a strains in around 2007. As of now, no update on novel circulating PCV-2 genotypes in swine has been published in Portugal, and no information on viral circulation in wild boar has ever been reported. To provide updated data on the PCV-2 genotypes currently circulating in Portugal, we screened wild boar stools for PCV-2, which were collected across Portugal during the 2018–2020 hunting seasons, and genetically characterized detected strains.

## 2. Materials and Methods

### 2.1. Sample Collection and Selection

Wild boar stool samples were collected opportunistically from October to February of 2018/2019 and 2019/2020, during the official hunting seasons. A total of 76 stools were collected from the posterior portions of the large intestine within 1–3 h post mortem. All animals were hunted for human consumption by professional hunters during legal game activities, hence no animals were killed for the purpose of this study. Sampling occurred in the central, central–western and southern regions of Portugal. All stool samples were frozen at −20 °C until testing.

### 2.2. Nucleic Acid Extraction

Stool suspensions (10%) were made in PBS pH 7.2 and centrifuged at 8000× *g* for 5 min. Viral extraction was carried out from 140 μL of clarified supernatants using the QIAcube^®^ automated platform (Qiagen, Hilden, Germany) and QIAamp DNA mini kit (Qiagen, Hilden, Germany), according to the manufacturer’s instructions. Eluted DNA was kept in RNase-free water at −80 °C.

### 2.3. PCV-2 Detection

Detection of PCV-2 was performed using previously described primers targeting the open reading frame 2 (*ORF2*; capsid protein gene), amplifying a 685 bp product [18]. Conventional PCR amplification was carried out using Xpert Fast Hotstart Mastermix (2X) with dye (Grisp, Porto, Portugal), according to the company’s protocol instructions. Briefly, amplification was carried out on 25 μL reaction mix containing 1 μL of each mentioned primer at 10 pmol/μL, 12.5 μL of Xpert Fast Hotstart Mastermix (2X) with dye, 5.5 μL PCR grade water and 5 μL of extracted DNA.

Amplification reactions, with the corresponding positive and negative (distilled water) controls, were conducted in Bio-Rad T100TM Thermal Cycler with the following conditions: initial cycle of 3 min at 95 °C (enzyme activation, denaturation of template DNA), followed by 40 cycles of 95 °C for 15 s, 53 °C for 15 s, and 72 °C for 2 s, with a final elongation at 72 °C for 10 min. In the end, PCR amplification products were electrophoresed at 100 V for 40 min on 1.5% agarose gel stained with Xpert Green Safe DNA gel stain (Grisp, Porto, Portugal), and then irradiated with UV light to identify the target DNA fragments. A DNA weight comparison was used for measurements (100 bp DNA ladder; Grisp, Porto, Portugal).

### 2.4. Sequencing and Phylogenetic Analysis

Presumptively positive amplicons of PCV-2 *ORF2* were purified with GRS PCR and Gel Band Purification Kit (Grisp, Porto, Portugal) and, using the Sanger method, bidirectional sequencing was performed with both specific primers of the target gene. Sequences were aligned with BioEdit Sequence Alignment Editor v7.1.9 software package, version 2.1 (Ibis Biosciences, Carlsbad, CA, USA) and compared with the sequences available in the NCBI (GenBank, Carlsbad, CA, USA) nucleotide database (http://blast.ncbi.nlm.nih.gov/Blast, accessed on 10 January 2022). Phylogenetic analysis was accomplished using MEGA version X software [19] and the Interactive Tree Of Life (iTOL) platform [20], with the capsid protein gene (*ORF2*) of PCV-2 sequences identified in this study and other representative sequences obtained from GenBank. This analysis was inferred using the maximum likelihood (ML) method [19,21]. The ML bootstrap values were estimated using 1000 replicates with Tamura 3-parameter model [21]. This model was estimated as the best substitution model by MEGA version X [19]. Sequences obtained in this study were then uploaded to GenBank.

## 3. Results

From a total of 76 stool samples of wild boar tested by PCR for the partial PCV-2 *ORF2* gene, two sequences were obtained (2/76; 2.6%, 95% confidence interval: 0.032–9.18). Both samples were from juvenile animals: a male from Évora (Cabeção parish: −8°2′59.172″, 38°56′22.7796″) and a female from Portalegre (Maranhão parish: −7°57′0.2412″, 38°59′43.5768″).

**Figure 1:**
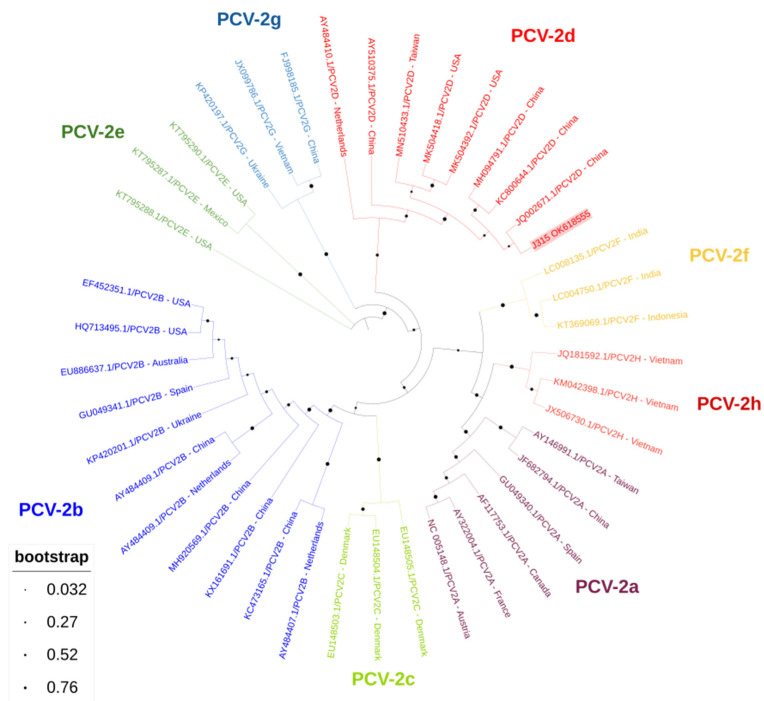
Phylogenetic tree inferred using the MEGA X maximum likelihood method (Tamura 3-parameter model) and the Interactive Tree of Life (iTOL) based on 41 nucleotide PCV-2 sequences, including J315 sequence obtained in this study (PCV-2d, plus its accession number, is in bold and shaded in red) and 40 strains of different genotypes obtained from GenBank (PCV-2a to PCV-2h) (no bold or shading and identified with the accession number and country of origin).

The amplified products obtained from these two wild boar stools were subjected to bidirectional sequencing, showing that the sequences were 100% identical to each other and both of the PCV-2d genotypes, after Basic Local Alignment Search Tool analyses. Further characterization by BLAST indicated that both sequences shared 100% identity with PCV-2 isolate sequences obtained from China (KY655968 and KX960933). One of the sequences spanned only 170 nt (and the other 658 nt), hence phylogenetic analysis was performed considering only the latter sequence to obtain more robust information about its genetic relatedness with other PCV-2 reference sequences (Figure 1). This confirmed the classification as PCV-2d. The following accession numbers were assigned to the sequences obtained: Accession numbers: OK618554, OK618555.

## 4. Discussion

The present study assessed the circulation of PCV-2 in wild boar in Portugal. This is the first description of PCV-2 in wild boar from this country. To the best of our knowledge, only one other study has detected and characterized PCV-2 in Portugal, showing viral circulation in domestic swine [17]. The study relied on sampling between 2003 and 2010 in Portugal and showed, after a genetic characterization, that the isolates belonged to PCV-2b genotype, speculating that the virus was introduced in Portugal around 2003 through the importation of live domestic swine from the Netherlands, Spain or France [17]. Data from the same study also suggested that PCV-2a strains were introduced in Portugal around 2007 but did not show the circulation of genotypes other than these. In the present study, two wild boar stool samples were shown to be positive for PCV-2 (2/76; 2.6%, 95% confidence interval: 0.032–9.18). PCV-2 fecal prevalence can be considered somewhat low when compared to previous studies conducted on swine from Germany (18.1%) [10], Belgium (35.6%) [11], the Czech Republic (43%) [13], Spain (23–58%) [15], Italy (10.9%) [14], Slovenia (25%) [12] and Hungary (20.5%) [16]. Compared to intensive pig breeding, these results are expected, since higher rates of infection tend to be associated with intensively managed populations in contrast to wild or extensively managed populations [15].

A BLAST analysis of detected PCV-2 strains and confirmed their classification as the PCV-2d genotype. Therefore, this is the first report describing not only the presence of PCV-2 in wild boars in Portugal but the first to report the PCV-2d genotype in the country. Interestingly, it was suggested that a key genotype change from the former PCV-2b to the latter PCV-2d is recent and occurs in some developing regions [22]. As such, this could be the case for PCV-2 in Portugal; this genotype has already been identified in China [8] and was recently suggested to be occurring in Italy [1]. Overall, with the recent documented genotype shift in other countries, evidence from this work suggests that a similar shift could be happening with PCV-2d in Portugal, eventually replacing other circulating genotypes in the coming years.

The PCV-2d genotype was first identified in China, although subsequent transboundary spread [8] was later questioned, since China is known for only importing (and not exporting) swine [23]. A possible explanation for viral spread was proposed, with wild boar being reservoirs that circulate freely and uncontrollably. However, the available information on wild boar’s role as a reservoir of PCV-2 is conflicting. Some authors agree that the wild boar reservoir is known to play a role in dissemination, particularly because their susceptibility to infection seems to be similar to that of pigs [11,15]. Accordingly, some authors even emphasize the significance of the interaction between these two species in disease maintenance [24,25]. Nevertheless, other researchers believe that this transmission is only theoretical and still controversial, as was previously suggested for other swine viruses such as African swine fever virus [26]. Although no direct evidence of PCV-2d genotype transmission between wild boar and domestic swine has been demonstrated, this theoretical transmission could still be the case, hence alerts should be made to the relevant veterinary authorities concerning veterinary public health. This is particularly important since porcine-circovirus-associated diseases are known to have a significant impact on animal production. In particular, estimates of PMWS costs in England and Europe reach circa GBP 53 and EUR 900 million per year [6,7]. The available vaccines are based on PCV-2a and PCV-2b genotypes, and recent data show the cross protection of these vaccines to existing PCV-2 genotypes [27]. As such, the circulation of PCV-2d, albeit of concern, could somehow become controllable with the existing vaccines. However, some reports show that novel genotypes are concerning, as new genetic variants with altered tropisms can eventually evade vaccination and cause subclinical illness, also leading to significant short- and long-term economic losses, or even significant morbidity.

## 5. Conclusions

Data from the present study, albeit reporting a low PCV-2 circulation in wild boar, can be considered relevant since the wild boar population in Portugal is known to be spread across the territory [28], inhabiting various ecological niches, from natural to humanized habitats, and can easily contact domestic swine, and thus become a vehicle for transmission. Monitoring wild PCV-2 reservoirs is important for both veterinary public health and economic reasons, since PCV-2 infection has a strong economic impact on the swine industry [7]. The monitoring of new emerging strains can assist in the mitigation of the spread of new genotypes and variants that could cause immunity failure.

## Data Availability

Not applicable.
